# Combination antiretroviral therapy and cell–cell spread of wild-type and drug-resistant human immunodeficiency virus-1

**DOI:** 10.1099/jgv.0.000728

**Published:** 2017-05-05

**Authors:** Boghuma Kabisen Titanji, Deenan Pillay, Clare Jolly

**Affiliations:** ^1^​ Division of Infection and Immunity, University College London, London WC1E 6BT, UK; ^2^​ Department of Medicine, Emory University School of Medicine, Atlanta, USA; ^3^​ Africa Centre for Health and Population Sciences, University of KwaZulu-Natal, KwaZulu-Natal, South Africa

**Keywords:** HIV, cell–cell, cART, T cell, drug resistance

## Abstract

Human immunodeficiency virus-1 (HIV-1) disseminates between T cells either by cell-free infection or by highly efficient direct cell–cell spread. The high local multiplicity that characterizes cell–cell infection causes variability in the effectiveness of antiretroviral drugs applied as single agents. Whereas protease inhibitors (PIs) are effective inhibitors of HIV-1 cell–cell and cell-free infection, some reverse transcriptase inhibitors (RTIs) show reduced potency; however, antiretrovirals are not administered as single agents and are used clinically as combination antiretroviral therapy (cART). Here we explored the efficacy of PI- and RTI-based cART against cell–cell spread of wild-type and drug-resistant HIV-1 strains. Using a quantitative assay to measure cell–cell spread of HIV-1 between T cells, we evaluated the efficacy of different clinically relevant drug combinations. We show that combining PIs and RTIs improves the potency of inhibition of HIV-1 and effectively blocks both cell-free and cell–cell spread. Combining drugs that alone are poor inhibitors of cell–cell spread markedly improves HIV-1 inhibition, demonstrating that clinically relevant combinations of ART can inhibit this mode of HIV-1 spread. Furthermore, comparison of wild-type and drug-resistant viruses reveals that PI- and RTI-resistant viruses have a replicative advantage over wild-type virus when spreading by cell–cell means in the presence of cART, suggesting that in the context of inadequate drug combinations or drug resistance, cell–cell spread could potentially allow for ongoing viral replication.

## Abbreviations

cART, combination antiretroviral therapy; CI, combination index; HIV-1, human immunodeficiency virus-1; NNRTI, non-nucleoside reverse transcriptase inhibitor; NRTI, nucleoside reverse transcriptase inhibitor; PI, protease inhibitor; qPCR, quantitative PCR; RTI, reverse transcriptase inhibitor; VS, viral synapse.

## Introduction

Over the last three decades, combination antiretroviral therapy (cART) has completely transformed the prognosis of human immunodeficiency virus 1 (HIV-1) infection from a fatal disease to a manageable chronic condition. Despite the success of cART, life-long treatment is limited by cost, development of drug resistance, the need for life-long adherence to therapy and the unknown effects of long-term treatment. In recent years, the focus of HIV-1 research has shifted towards the search for a definitive cure for HIV/AIDS alongside ongoing efforts to develop a vaccine to prevent new infections. These strategies are, however, far from being realized and antiretroviral drugs remain the best weapon to treat and potentially prevent HIV-1 infection. cART leads to a dramatic suppression of viral replication, reducing the plasma levels of circulating HIV-1 RNA copies below levels detectable by conventional clinical assays [1–4]. Despite this considerable success, patients on cART have episodes of intermittent detectable HIV-1 RNA in plasma (blips) [5–7], viral rebound with therapy interruption and can develop antiretroviral drug resistance [4]. Several factors have been hypothesized to explain the inability to completely eradicate HIV-1 using cART, including reduced susceptibility of HIV-1 to cART during cell–cell spread that may contribute to ongoing low-level viral replication [8].

HIV-1 can disseminate between cells either by cell-free fluid phase diffusion of budded viral particles, or by direct cell–cell spread. Cell–cell spread of HIV-1 across a virological synapse (VS) [9] is a highly efficient mode of viral dissemination in comparison to the classical mode of cell-free diffusion [9–14]. This is mainly attributable to the polarized budding of the virus towards the target cell, close physical contact between the effector and target cell, and the clustering of cellular receptors and viral envelope proteins at the interface between the effector cell and the target cell [9, 10, 14, 15]. These factors limit the need for prolonged diffusion and facilitate the efficient transfer of viral particles from the donor cell to the target cell [9, 10, 14, 15]. Numerous studies have demonstrated the reduced susceptibility of cell–cell spread to the effects of some neutralizing antibodies [10, 11, 16–18] and more recently to some classes of antiretroviral drugs [8, 19–22]. Indeed, we and others have shown the reduced efficacy of some classes of antiretroviral drugs applied as single agents [8, 19–21]. While protease inhibitors (PIs) remain effective inhibitors of both HIV-1 cell–cell and cell-free infection, some reverse transcriptase inhibitors (RTIs) have significantly reduced potencies against the former mode of infection [19]. These observations seem paradoxical given the strong evidence that cART effectively inhibits viral replication in HIV-1-infected patients [4]. However, conventional first-line cART typically consists of three antiretroviral drugs from at least two different therapeutic classes [4, 23, 24], rather than administration of a single agent. Although two nucleoside RTIs (NRTIs) usually form the backbone of the triple-drug regimen, the third component can be a non-NRTI (NNRTI), a PI or an integrase inhibitor (INI) [23, 24]. Whether clinically relevant drug combinations suppress cell–cell spread of HIV-1 remains unknown, but the matter has clear implications for antiretroviral treatment and eradication strategies.

In the present study, we explore the impact of clinically relevant PI- and RTI-based combinations against cell–cell spread of wild-type and drug-resistant HIV-1 and additionally consider interactions of drugs delivered in combination [25–27]. Our results show that PI- and RTI-based antiretroviral drug combinations effectively inhibit both cell–cell and cell-free spread of HIV-1. Combining drugs that alone are poor inhibitors of cell–cell spread markedly improves HIV-1 inhibition, demonstrating that clinically relevant drug combinations can suppress HIV-1 replication mediated by highly efficient cell–cell spread. We also report that relatively less fit drug-resistant viruses regain a replicative advantage when spreading by a cell–cell mechanism in the presence of cART, suggesting that in a context of inadequate cART or drug resistance, cell–cell spread may contribute to treatment failure and viral replication.

## Results

### RTI-based combination therapies effectively inhibit cell–cell and cell-free spread of HIV-1

We and others have previously shown that the RTIs, Tenofovir (TFV), ﻿Zidovudine (AZT), ﻿Lamivudine (3TC) and ﻿Nevirapine (NVP), have significantly reduced potency against HIV-1 cell–cell infection when compared to cell-free infection [8, 19–21]. Importantly, these drugs are not used as monotherapy for the treatment of HIV-1-infected patients, due to the risk of rapidly selecting for drug-resistant variants of the virus. To evaluate the efficacy of clinically relevant drug combinations, we combined different drugs used in cART and measured their activity against HIV-1 cell–cell spread. To do this we took two approaches. Firstly, we simply compared whether drug combinations were able to fully suppress cell–cell spread *in vitro*. Secondly, we considered whether, when combined, different drug combinations showed improved potency (i.e. synergistic or additive effects) against cell–cell spread. For this, we applied the median effect analysis based on the median effect principle of Chou and Talalay [25–27] to determine the combination index and define whether the interactions between the drugs in the combinations tested were additive, synergistic or antagonistic and compared this between cell–cell and cell-free modes of HIV-1 infection. The median effect analysis is a well-established method for assessing drug interactions and has been applied extensively for evaluating combinations of antiretroviral drugs in drug development studies [28–36].

A well-established quantitative PCR (qPCR) assay for HIV-1 reverse transcripts that directly measures the early steps of HIV-1 infection [10, 19, 37] was used to measure cell–cell and cell-free spread in the presence of RTI-based combinations. This assay was previously used to test the efficacy of PIs and RTIs against HIV-1 cell–cell spread [19]. Having previously validated that Jurkat T cells and primary T cells give similar results when studying cell–cell spread in the presence of ART [19], the Jurkat T cell model system was used here due to the large number of cells needed and the complexity of multiple drug comparisons. Initially, three dual combinations of RTIs – AZT+TFV, TFV+EFV(Efavirenz) and AZT+NVP – were tested. These were chosen as we and others have previously shown that these drugs, when used as single agents, are poor inhibitors of cell–cell spread, compared to PIs for example [8, 19–21]. The drugs were combined in a fixed-dose ratio based on their IC_50_ values determined in a cell-free infection assay [25–27, 38]. Cell–cell and cell-free spread of wild-type HIV-1 (HIV-1wt) was measured in the presence of a serial dilution of this fixed dose combination. The inhibitory effect (*f*_a_) of each drug alone and as part of a combination was calculated and expressed as a fraction, representing inhibition of infection in the presence of the drug relative to the no-drug control. These *f*_a_ values were inputted in CompuSyn to determine the combination index (CI) for different concentrations of the drug combination. The mean CI values for 50, 75, 90 and 95 % inhibition levels obtained from two independent experiments and the standard error of the mean are presented.

**Fig. 1.**
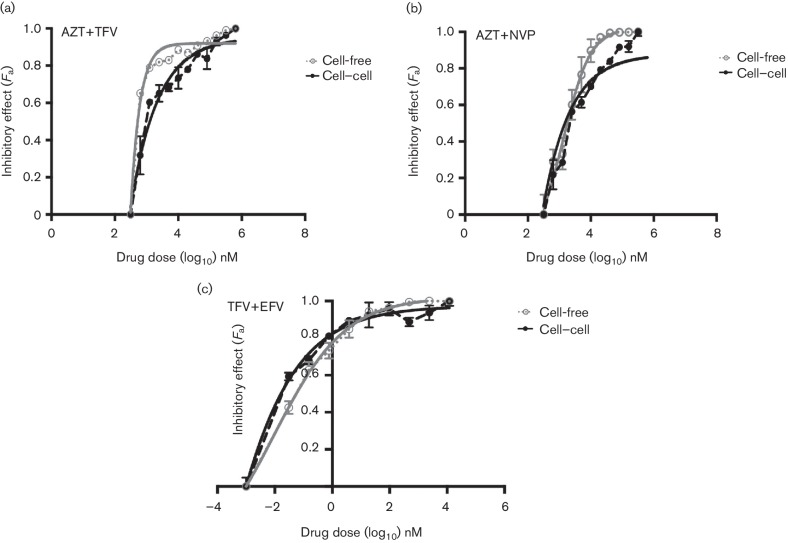
RTI-based cART effectively inhibits cell-to-cell and cell-free spread of HIV-1. Cell-to-cell and cell-free infections were assessed in the presence of a serial dilution of dual RTI-based drug combinations: (a) AZT+TFV, (b) AZT+NVP and (c) TFV+EFV. The drugs were combined in ratios based on their IC_50_ values determined in a cell-free infection assay. Infection was quantified by detecting HIV-1 *pol* DNA transcripts generated at each dilution of the combination by qPCR and expressed as a fraction of the no drug control. A representative from two independent experiments is shown. The error bars represent the standard deviation of the mean. The bold lines represent the non-linear regression curve-fit and dotted lines represent actual data points.

TFV and AZT, which we have previously shown to be respectively >10- and >20-fold less potent against cell–cell HIV-1 infection, were tested in combination against both cell–cell and cell-free infections. Fig. 1(a) shows that while this combination was able to block both modes of viral spread, more drug was needed to cause equivalent inhibition of cell–cell spread compared to cell-free. CI values showed additive/synergistic effects against cell–cell infection and synergistic effects against cell-free infection (Table 1). AZT and TFV were then respectively combined with non-nucleoside inhibitors NVP and EFV to reflect clinically relevant combinations (Fig. 1b, c). Both AZT and EFV drugs are frequently administered in combination with NRTIs in first-line cART regimens. In our hands, NVP as a single agent has a four-fold reduced potency against cell–cell spread of HIV-1 [19] whereas EFV is equally potent against both cell–cell and cell-free spread of HIV-1, as for PIs [39]. The combination of AZT+NVP inhibited both cell–cell and cell-free modes of HIV-1 spread with synergistic/additive effects against cell–cell infection and synergistic effects against cell-free infection (Fig. 1b and Table 1). Again, we did observe a slight reduction in efficacy of this drug combination against cell–cell spread compared to cell-free. Notably, TFV+EFV was found to be the most effective combination and potently inhibited both cell-free and cell–cell spread at low concentrations (Fig. 1c). For all the RTI-based combinations tested above, we consistently observed a stronger combined potency of the drugs against cell-free infections compared to cell–cell infections, indicative somewhat of a reduced drug activity during cell–cell spread.

**Table 1.** Combination indices for cell–cell and cell-free HIV-1 spread

Mode of infection		Combination index (CI)*†				Effect
		50	75	90	95	
	Combination AZT+TFV (ratio=1 : 1)					
Cell–cell	AZT+TFV	0.9 (0.1)	0.85 (0.1)	0.82 (0.05)	0.74 (0.12)	Additive/synergistic
Cell-free	AZT+TFV	0.41 (0.06)	0.39 (0.03)	0.42 (0.1)	0.45 (0.04)	Synergistic
	Combination AZT+NVP (ratio=40 : 1)					
Cell–cell	AZT+NVP	0.95 (0.05)	1.05 (0.05)	1.1 (0.13)	1.1 (0.1)	Additive
Cell-free	AZT+NVP	0.97 (0.06)	0.89 (0.04)	0.79 (0.06)	0.76 (0.1)	Additive/synergistic
	Combination TFV+EFV (ratio=1000 : 1)					
Cell–cell	TFV+EFV	0.59 (0.05)	0.46 (0.07)	0.36 (0.05)	0.35 (0.02)	Synergistic
Cell-free	TFV+EFV	0.1 (0.02)	0.13 (0.01)	0.22 (0.05)	0.39 (0.1)	Synergistic
	Combination LPV+TFV (ratio=1 : 1000)					
Cell–cell	LPV+TFV	0.03 (0.06)	0.1 (0.01)	0.12 (0.04)	0.36 (0.2)	Synergistic
Cell-free	LPV+TFV	0.02 (0.01)	0.06 (0.01)	0.15 (0.02)	0.4 (0.13)	Synergistic
	Combination LPV+NVP (ratio=1 : 25)					
Cell–cell	LPV+NVP	1.1 (0.07)	0.92 (0.2)	0.74 (0.24)	0.71 (0.26)	Additive/synergistic
Cell-free	LPV+NVP	0.86 (0.03)	0.72 (0.03)	0.6 (0.04)	0.46 (0.04)	Additive/synergistic

*Data are mean and sem from two independent experiments.

†50, 75, 90 and 95 indicate the percentage inhibition of infection in the presence of the combination being tested.

### PI-based combination therapies effectively inhibit cell–cell and cell-free spread of HIV-1

**Fig. 2.**
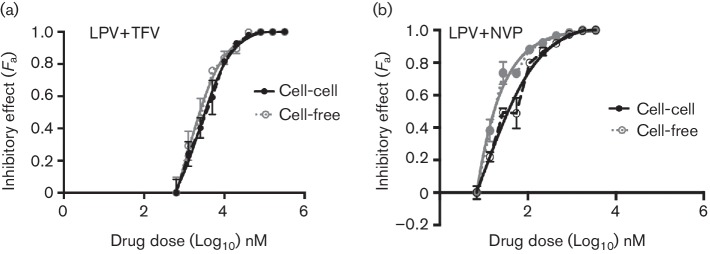
PI-based cART effectively inhibits both cell-to-cell and cell-free spread of HIV-1. Cell-to-cell and cell-free infections were assessed in the presence of a serial dilution of a PI+RTI combination: (a) LPV+TFV and (b) LPV+NVP. The drugs were combined in a ratio based on their IC_50_ values determined in a cell-free infection assay. Infection was quantified by detecting HIV-1 *pol* DNA transcripts generated at each dilution of the combination by qPCR and expressed as a fraction of the no drug control. A representative from two independent experiments is shown. The error bars represent the standard deviation of the mean. The bold lines represent the non-linear regression curve-fit and dotted lines represent actual data points.

In contrast to RTIs, PIs are equally effective against both cell–cell and cell-free spread of HIV-1 [19]. The introduction of PIs in the mid-1990s completely revolutionized cART and these drugs are now important components of both first-line and second-line treatment options [4, 23, 24]. We therefore wanted to explore the effects of combining the less effective RTIs with PIs against HIV-1 spread. The PI Lopinavir (LPV) was tested in combination with the NRTI TFV and in combination with the NNRTI NVP. We opted to use LPV as opposed to other PIs because of its position as first-line PI therapy in many resource-limited high-prevalence countries. Both combinations, LPV+TFV and LPV+NVP, potently inhibited cell–cell and cell-free spread of HIV-1 (Fig. 2a, b and Table 1).

### Drug-resistant viruses gain a replicative advantage when spreading cell–cell in the presence of cART

The development of drug resistance remains one of the biggest challenges of cART. Cell–cell spread of drug-resistant viruses and its possible implications for cART is therefore important. To study the interplay between drug resistance and cell–cell spread of HIV-1 in the context of dual and triple ART combinations, we tested PI and RTI drug-resistant viruses commonly selected by cART *in vivo* and *in vitro*. The PI-resistant virus carrying the V82A mutation in protease and the compensatory A431V mutation in Gag (HIV-1dm) is selected for by LPV therapy [40–44] and confers varying degrees of resistance to all PIs except DRV [43, 44]. The NRTI-resistant virus (HIV-1m184v) with M184V in RT is selected for by suboptimal ART containing 3TC or FTC and confers a high level of resistance to these two agents [45, 46]. The NNRTI-resistant virus (HIV-1k103n) with K103N in RT is selected for by NVP or EFV containing ART and confers high levels of resistance to these drugs [47–49]. Besides conferring resistance to the relevant antiretroviral agents, these mutations impose a fitness cost for the replication of these viruses when compared to wild-type HIV in cell-free assays [40, 50, 51].

**Fig. 3.**
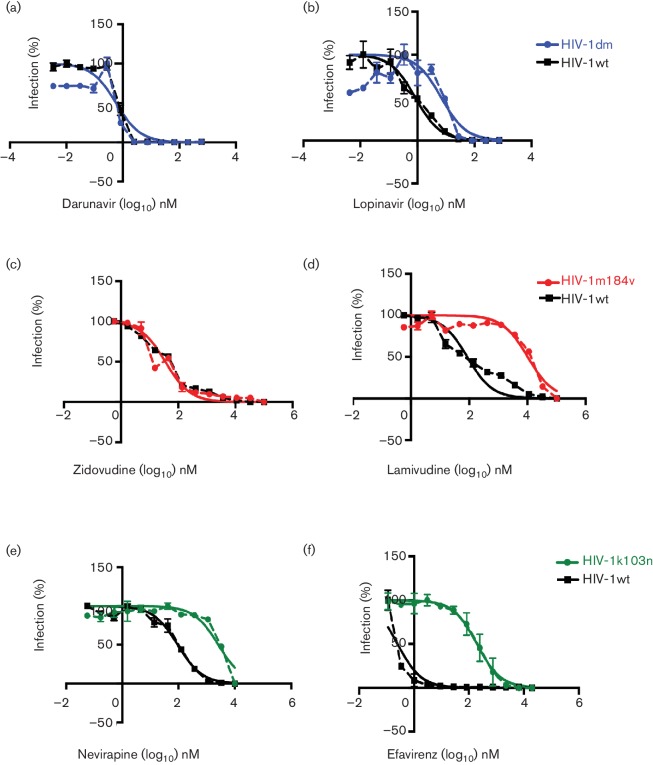
Drug susceptibility of drug-resistant viruses compared to wild-type HIV-1. Drug susceptibility of the drug-resistant viruses was tested in a cell-free-based HeLa TZM-bl drug susceptibility assay. The phenotypes of the viruses were confirmed in this assay and compared to wild-type virus: (a) HIV-1dm_D_ was 8.4-fold more resistant to LPV than HIV-1wt but was (b) equally susceptible to DRV as HIV-1wt. (c) HIV-1m184v was as expected susceptible to AZT but was (d) 120-fold more resistant to 3TC than HIV-1wt. (e) HIV-1k103n was 28-fold more resistant to NVP than HIV-1wt, and was (f) 650-fold more resistant to EFV that HIV-1wt. The dotted lines represent actual data points while the bold lines represent the non-linear regression curve fit. The error bars represent the standard deviation of the mean and a representative experiment of two independent repeats is shown.

**Fig. 4.**
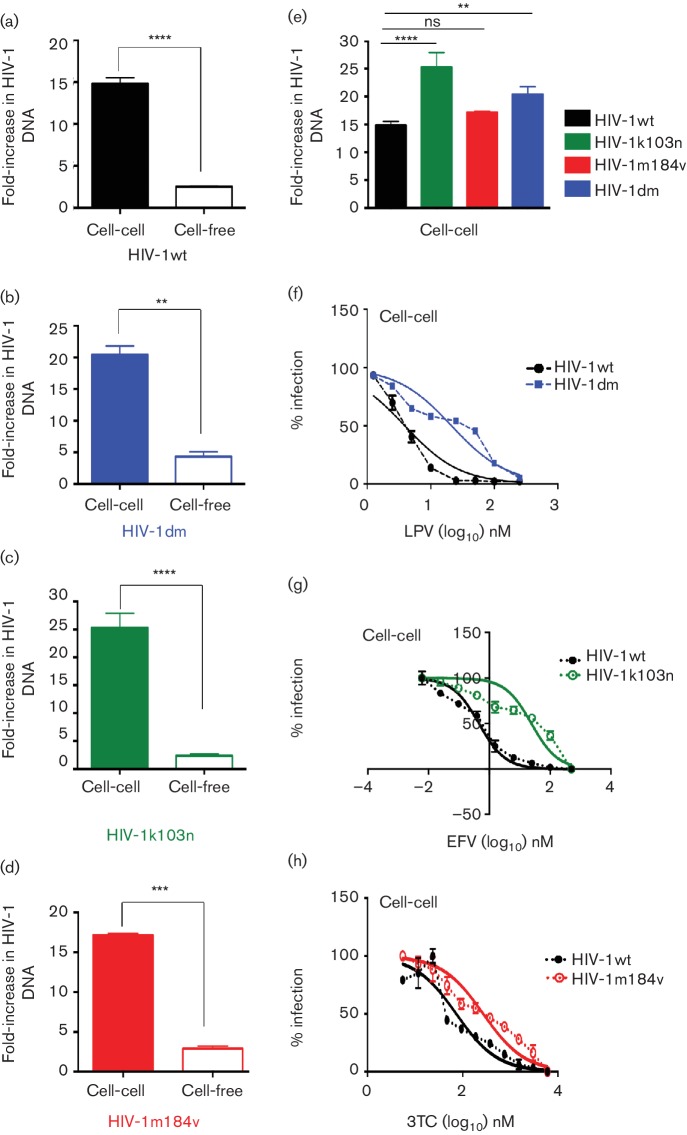
Drug-resistant viruses spread efficiently by a cell-to-cell mechanism and maintain their resistant phenotype with this mode of spread. Cell-to-cell spread of (a) HIV-1wt, (b) HIV-1dm, (c) HIV-1k103n and (d) HIV-1m184v was measured and compared to their cell-free spread using a qPCR-based cell-to-cell assay system. All viruses spread efficiently by a cell-to-cell mechanism, with this mode of spread being 5–7 times more efficient for all viruses tested in comparison to cell-free spread. (e) Drug-resistant viruses spread from cell to cell with comparable efficiency to wild-type virus. Drug-resistant viruses (f) HIV-1dm, (g) HIV-1k103n and (h) HIV-1m184v all maintain their resistant phenotype when spreading by a cell-to-cell mechanism. A representative of two independent repeats is shown. Error bars show the standard deviation of the mean. Statistical comparisons were done using a paired Student *t*-test. ****P<0.0001, ***P<0.001, **P<0.01, ns not significant.

The drug susceptibility phenotypes of the resistant viruses compared to HIV-1wt were verified in an in-house drug susceptibility assay [52, 53]. As expected, HIV-1dm was susceptible to inhibition by DRV but was 8.4-fold more resistant to inhibition by LPV (Fig. 3a, b). HIV-1m184v was susceptible to inhibition by AZT but was 120-fold more resistant to inhibition by lamivudine (3TC) (Fig. 3c, d). HIV-1k103n was 28-fold more resistant to inhibition by NVP and 650-fold more resistant to inhibition by EFV. After verifying and confirming the phenotype of drug-resistant viruses, the ability of these viruses to spread efficiently by a cell–cell compared to a cell-free mechanism was assessed using the qPCR-based assay system. HIV-1wt cell–cell spread was significantly more efficient (six-fold) than cell-free spread (Fig. 4a), in agreement with previous reports [10–14, 37, 54]. Similarly, all drug-resistant viruses tested showed more efficient cell–cell spread compared to cell-free spread (Fig. 4b–d). As expected, all drug-resistant viruses maintained their resistant phenotypes when spreading by a cell–cell mechanism and unlike wild-type virus were not inhibited by the presence of the relevant anti-retroviral drug (Fig. 4f–h).

**Fig. 5.**
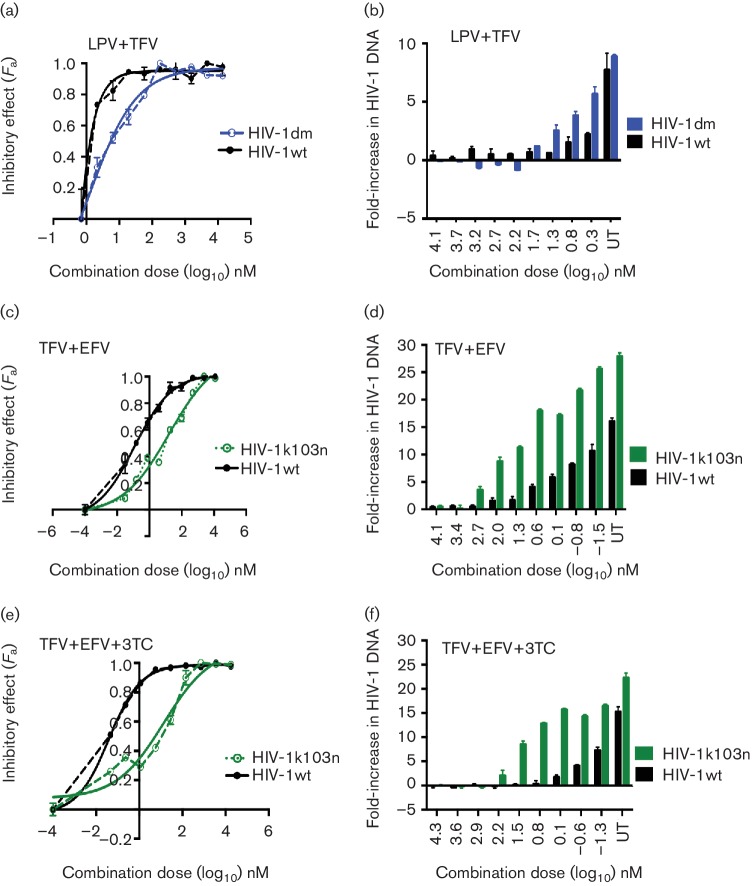
Drug-resistant viruses gain a replicative advantage when spreading by a cell-to-cell mechanism in the presence of cART. Cell-to-cell spread of drug-resistant viruses was compared to wild-type virus in the presence of a serial dilution of fixed-dosed PI- and RTI-based drug combinations. Infection was quantified by qPCR detection of HIV-1 *pol* DNA. HIV-1dm spreads more efficiently by a cell-to-cell mechanism compared to wild-type virus in the presence of LPV+TFV (a, b). HIV-1k103n spreads efficiently by a cell-to-cell mechanism compared to wild-type virus in the presence of TFV+EFV (c, d) and in the presence of TFV+EFV+3TC (e, f). A representative experiment of two independent repeats is shown. The error bars represent the standard deviation of the mean; UT, untreated.

Next, we assessed cell–cell spread of the resistant viruses in the presence of combination therapy. We tested the PI-resistant virus HIV-1dm in the presence of PI-based cART and the RTI-resistant viruses HIV-1k103n and HIV-1m184v in the presence of RTI-based cART. To evaluate the impact of drug resistance on the potency of a given combination, we calculated the CI values for the drug-resistant viruses and compared them to those obtained with the wild-type virus. With the combination of LPV+TFV, HIV-1dm (which is resistant to LPV) had a replicative advantage compared to HIV-1wt when spreading by a cell–cell mechanism (Fig. 5a, b and Table 2), indicating that TFV alone is unable to suppress cell–cell spread, in agreement with previous reports [8, 19–21]. A similar result was obtained for cell–cell spread of HIV-1k103n compared to HIV-1wt in the presence of TFV+EFV, with the resistant virus again showing a distinct replicative advantage over wild-type virus (Fig. 5c, d and Table 2). These results suggest that cell–cell spread of drug-resistant viruses reduces the potency of cART. Combinations that are synergistic against cell–cell spread of wild-type virus become additive or exhibit decreased synergy against cell–cell spread of drug-resistant viruses (Table 2). This observation remained apparent even when TFV+3TC+EFV, a potent clinically relevant first-line triple drug combination, was tested against cell–cell spread of HIV-1k103n (Fig. 5e, f and Table 2).

**Table 2.** CI values against PI-resistant virus (HIV-1_DM_), RTI-resistant virus (HIV-1_K103N_) and wild-type virus during cell-cell spread

Virus		Combination index (CI)*†				Effect
		50	75	90	95	
	Combination (ratio=1 : 1000)					
HIV-1wt	LPV+TFV	0.03 (0.06)	0.1 (0.01)	0.12 (0.04)	0.36 (0.2)	Synergistic
HIV-1dm	LPV+TFV	0.14 (0.01)	0.21 (0.01)	0.29 (0.04)	0.49 (0.1)	Synergistic
	Combination TFV+EFV (ratio=1000 : 1)					
HIV-1wt	TFV+EFV	0.69 (0.05)	0.46 (0.07)	0.36 (0.05)	0.35 (0.02)	Synergistic
HIV-1k103n	TFV+EFV	1.1 (0.02)	0.93 (0.01)	0.82 (0.05)	0.79 (0.1)	Synergistic/additive
	Combination TFV+EFV+3TC (1000 : 10 : 1)					
HIV-1wt	TFV+3TC+EFV	0.08 (0.03)	0.09 (0.01)	0.12 (0.04)	0.18 (0.01)	Synergistic
HIV-1k103n	TFV+3TC+EFV	0.35 (0.02)	0.39 (0.01)	0.44 (0.02)	0.48 (0.13)	Synergistic

*Data are mean and standard sem from two independent experiments.

†50, 75, 90 and 95 indicate the percentage inhibition of infection in the presence of the combination being tested.

**Fig. 6.**
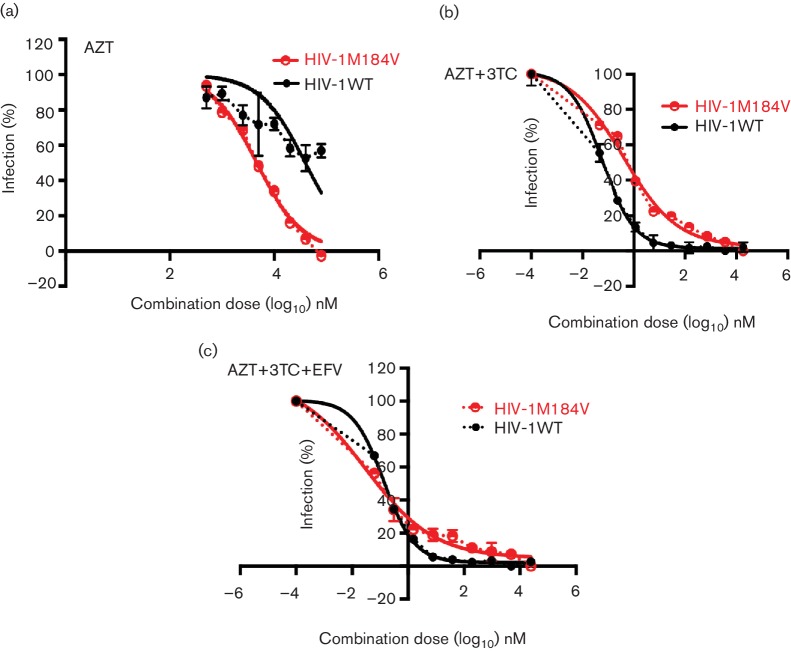
M184V mutation restores susceptibility to AZT and cART during cell-to-cell spread of HIV-1. Cell-to-cell spread of NRTI mutant HIV-1m184V compared to cell-to-cell spread of HIV-1wt in the presence of RTI mono (a), dual (b) and triple therapy (c). (a) AZT effectively inhibits cell-to-cell spread of HIV-1m184v, although it is ineffective against cell-to-cell spread of HIV-1wt. (b) In the presence of a serial dilution of a fixed dose combination of AZT+3TC, cell-to-cell spread of HIV-1wt and HIV-1m184v are effectively blocked although HIV-1m184v has a replicative advantage over wild-type virus. (c) Triple combination of AZT+3TC+EFV potently inhibits cell-to-cell spread of both HIV-1wt and HIV-1m184v. The error bars represent the standard deviation of the mean and a representative from two independent experiments is shown. The bold lines represent the non-linear regression curve-fit and dotted lines represent actual data points.

Finally, cell–cell spread of the NRTI drug-resistant virus HIV-1m184v was tested in the presence of an RTI-based combination and compared to HIV-1wt. The M184V mutation in RT was particularly interesting for this study because while it confers a 120-fold greater resistance to inhibition by 3TC compared to wild-type virus in the drug susceptibility assay (Fig. 3d), this mutation is also well described for increasing the susceptibility of the virus to other NRTIs, notably TFV and AZT [55–58]. It was therefore of interest to test whether the increased susceptibility of HIV-1m184v to AZT would remain evident when infection was mediated by highly efficient cell–cell spread, not least because cell–cell spread by wild-type HIV-1 is highly impervious to inhibition by AZT [19]. To address this, cell–cell spread of the mutant virus was directly compared to that of the wild-type virus in the presence of AZT. Fig. 6(a) shows that although AZT was unable to fully suppress cell–cell spread of HIV-1wt, the drug effectively inhibited cell–cell spread of HIV-1m184v. Cell–cell spread of this resistant mutant was then tested with drug combinations. With the combination of 3TC+AZT, both HIV-1wt and HIV-1m184v cell–cell infections were effectively inhibited although HIV-1m184v showed a replication advantage albeit a modest one in the presence of this combination in comparison to HIV-1wt (Fig. 6b). The triple RTI combination of 3TC+AZT+EFV potently blocked cell–cell spread of both viruses (Fig. 6c). This finding confirmed the continued efficiency of clinically relevant triple cART in the presence of the M184V mutation.

## Discussion

In recent years, the role of HIV-1 cell–cell spread as a means of antiviral escape and a possible mechanism for the maintenance of the viral reservoir has elicited great interest and debate. Our group and others have demonstrated the variable effects of individual antiretroviral drug classes against this mode of infection *in vitro* [8, 19–21]. However, given the widely accepted and proven efficacy of cART for the treatment of HIV-infected patients, this has been a topic of much discussion.

Here we have assessed the potency of clinically relevant RTI and, for the first time, PI-based drug combinations against cell–cell spread of HIV-1 and compared this to the classical mode of infection by cell-free diffusion. We find cART potently inhibits both cell–cell and cell-free modes of viral dissemination, albeit with a moderately reduced potency against cell–cell infection that is a more efficient means of HIV-1 spread. This is further reflected by weaker observed combined effects (additive or synergistic) of the combinations tested against cell–cell infection, compared to cell-free infection, despite efficient suppression of viral dissemination *in vitro*. Notably, combining less effective RTIs (AZT+TFV, TFV+EFV, AZT+NVP) and RTIs with potent PIs (LPV+TFV, LPV+NVP) strongly inhibits cell–cell spread. These drug interactions are similar to those observed in previous cell-free-based antiviral drug interaction studies [29–32, 34, 35, 59, 60]. The reduced potency of some antiretrovirals against cell–cell spread has been mainly attributed to the high viral m.o.i. that characterizes this mode of dissemination *in vitro*. Thus it has been suggested that RTIs, which act within target cells, may be more easily saturated by an influx of infectious virions transmitted at the VS, explaining their reduced potency against cell–cell spread *in vitro* [8, 19, 21]. Our data showing that antiretroviral drugs display enhanced potency when used in combination suggest that cART is probably sufficient to overcome the high multiplicity of cell–cell infections in this *in vitro* model. Our data are supported by Agosto *et al.* [21] who evaluated inhibition of HIV-1 cell–cell spread in the presence of RTI combinations using the instantaneous inhibitory potential (IIP) as a parameter to assess the potency and inhibitory capacity of drugs in combination. Like the CI, the IIP is also derived from the median effect equation [25–27, 61, 62]. That two independent studies using different analytical approaches concur that cART can effectively block HIV-1 cell–cell spread addresses the important issue of how cART could control viral replication *in vivo*, in light of reports attesting to reduced efficacy of single agents against highly efficient cell–cell spread [8, 19–21]. Furthermore, here we have also included for the first time PIs in cART to evaluate the effect against cell–cell spread, as well as combinations with PIs and NNRTIs. While PIs and NNRTIs are not typically used in cART, that they also demonstrate potency against cell–cell spread further highlights the advantage of combining drugs, even those that alone are poor inhibitors of cell–cell spread, to achieve efficient viral blockade. Taken together, these studies demonstrate that different combinations of cART can effectively block cell–cell spread of HIV-1.

Drug resistance is a major cause of treatment failure, but the interplay between cell–cell infection and cART in the context of resistant virus has been little considered, but has important clinical implications [63, 64]. Our findings that drug-resistant viruses can spread effectively by a cell–cell mechanism in the presence of cART is therefore interesting. The reduced replication fitness of the M184V mutation and its enhanced susceptibility to Tenofovir and Zidovudine has been used as an argument for maintaining patients who select for this resistance mutation on 3TC containing treatment regimens [50, 51, 58, 65]. The rationale is that selecting for and maintaining the relatively unfit M184V virus will translate into a clinical benefit [50, 51, 58, 65]. While this approach appears to work initially, patients eventually fail treatment and still require the replacement of 3TC with alternative second-line options [66]. Here we observed that the NRTI drug-resistant mutant M184V, which has been extensively described in the literature for its diminished replication capacity in cell-free assays [50, 51, 55–57], was capable of cell–cell spread at levels similar to wild-type virus. Indeed, a similar observation has recently been made with the Dolutegravir (DTG)-resistant mutant R263K, which has a diminished capacity to replicate [67] but was found to spread efficiently via cell–cell contacts [68]. In light of our findings, and those of Agosto *et al.* [21], further testing to elucidate whether cell–cell spread may serve as a means for continued replication and maintenance of ‘unfit’ drug-resistant viruses and analysis of the mechanism involved would be of clear interest.

While it is difficult to extrapolate from *in vitro* models to explain the complexities of viral replication *in vivo*, it has been suggested that if cell–cell spread of HIV-1 is taking place in lymphoid tissue *in vivo*, then this should result in the appearance of cells with multiply-integrated proviruses [54]. Since only a few studies have demonstrated the presence of multiply infected CD4+ lymphocytes *in vivo* [69, 70], it has been argued that cell–cell spread does not contribute to HIV-1 replication *in vivo*. However, this may be an over-simplification. Firstly, it is increasingly apparent that the defensive processes associated with high m.o.i. infection induce cellular apoptosis as the result of a high cytoplasmic load of viral DNA [71–73]. This may in turn result in the selection of CD4+ lymphocytes carrying only a single provirus, which are more frequently observed *in vivo.* Thus these cells may simply be deleted from the pool of cells available for sampling. Secondly, it may not naturally follow that the attachment and entry of multiple viral particles would necessarily result in multiple infectious units that all lead equally to successful viral integration in the setting of natural spread *in vivo*. Many aspects of the complex molecular processes that regulate HIV-1 nuclear import and integration remain to be elucidated and future work will undoubtedly shed light on this issue.

Some studies have failed to show evidence of viral evolution in patients receiving fully suppressive cART, arguing against ongoing viral replication in treated patients [74–77]; however, due to the difficulty in sampling niches and sanctuary sites where cell–cell spread probably occurs, these data may not fully capture the true picture. Furthermore, recent data from some raltegravir intensification studies provide support for ongoing viral replication during cART [78–80], although these too are subject to debate (reviewed in [81]). *In vivo* HIV cell–cell spread would probably occur predominantly in the lymphoid tissues where there is an abundance of CD4+ T cells and effective antiviral drug penetration may be suboptimal [82]. Also, in other anatomical sanctuary sites with reduced drug penetration [82–85], niches of cells in close proximity and the absence of sheer flow may favor cell–cell spread. It is reasonable to speculate based on our results that in a context of low adherence to cART, sub-optimal drug combinations and drug resistance, cell–cell spread may allow for therapeutic escape due to its enhanced replication efficiency over cell-free spread, even in the presence of cART. The development of improved methods to identify and sample sanctuary sites in HIV-1-infected individuals will help to clarify this issue.

Predicting the outcomes of ART in patients, although highly desirable, remains very difficult in current clinical practice. As new therapies are developed for the treatment of HIV-1, being able to assess the efficacy of novel combinations against all modes of virus dissemination will serve as a valuable tool for predicting their efficacy, prior to clinical testing. In view of our findings, the variable effects of antiretroviral drugs on cell–cell spread of HIV-1 should be considered for future prophylactic, therapeutic and eradication strategies. Employing a simple *in vitro* assay like the one used for this study provides a straightforward way of doing this.

## Methods

### Cells, viruses and inhibitors

HeLa-TZMbl cells were obtained from the Center for AIDS Reagents, National Institutes of Biological Standard and Control, UK (CFAR, NIBSC) and donated by J. Kappes, X. Wu and Tranzyme Inc. HEK 293T cells were originally from the ATCC (American Type Culture Collection). Adherent cells were cultured in Dulbecco’s modified Eagle medium (DMEM) supplemented with 10 % FCS (Invitrogen), 50 U penicillin ml^−1^ and 50 µg streptomycin ml^−1^. The CD4+/CXCR4+ T cell line Jurkat CE6.1 [obtained through AIDS Research and Reference Reagent Program, Division of AIDS, NIAID, NIH (ARRP): from Dr Estuardo Aguilar-Cordova and Dr John Belmont] was maintained in RPMI 1640 supplemented with 10 % FCS and 50 U penicillin ml^−1^ and 50 µg streptomycin ml^−1^. The HIV-1 clone pNL4.3 was produced by Dr Malcolm Martin and obtained from the ARRP. The antiretroviral drugs Lopinavir (LPV), Nevirapine (NVP), Efavirenz (EFV), Zidovudine (AZT), Lamivudine (3TC) and Tenofovir (TDF) were obtained from the ARRP.

### Construction of drug-resistant viruses

To construct the drug-resistant variants of NL43, site-directed mutagenesis was performed with Accuprime Pfx supermix (Invitrogen) using forward and reverse primers containing the required nucleotide substitutions. For the PI-resistant virus (HIV-1dm) two mutations, V82A, a major protease drug resistance mutation (mutagenesis primers: forward 5′-GTAGGACCTACACCTGCCAACATAATTGGAAG-3′, reverse 5′-CAGATTTCTTCCAATTATGTTGGCAGGTGTAGG-3′), and A431V, a cleavage site mutation in the p7/p1 junction (mutagenesis primers: forward 5′-GAAAGATTGTACTGAGAGAGACAGGTTAATT TTTTAGG-3′, reverse 5′-GGCCAGATCTTCCCTAAAAATTAACCTGTCTCTCAGT-3′), were introduced. For the NRTI-resistant virus (HIVm184v), the M184V mutation was introduced in RT (mutagenesis primers: forward 5′-TCTATCAATACGTGGATGATTTGTATGTAGGATCTGACT TAG-3′, reverse 5′-AATCATCCACGTATTGATAGATGACTATGTCTGGATTTTG-3′). For the NNRTI-resistant virus (HIV-1k103n), the K103N mutation was introduced in RT (mutagenesis primers: forward 5′-GCAGGGTTAAAACAGAACAAATCAGTAACAGTACTGG-3′, reverse 5′-ACAT CCAGTACTGTTACTGATTTGTTCTGTTTTAAC-3′). The mutagenesis was carried out in the vector pCR 2.1 TOPO, by sub-cloning a region of HIV-1NL4.3 covering nucleotides 740–2940. Sequencing was performed by BigDye terminator chemistry and a 3730xl analyzer (ABI) to confirm the presence of the mutations introduced and the absence of any other substitutions. The mutated fragment was re-introduced into the HIV-1NL4.3 backbone by *Spe*I/*Age*I digestion. Stocks of infectious virus (HIV-1dm, HIV-1m184v, HIV-1k103n and HIV-1wt) were made by transfecting 293T cells using Fugene HD (Promega). Infectious viral titres were measured on HeLa-TZMbl reporter cells using the Bright-Glo Luciferase assay kit (Promega).

### Drug susceptibility assay to validate mutagenesis

An in-house assay was used to determine the drug susceptibility of the mutant drug-resistant viruses compared to the wild-type vector [52, 53]. The assay was modified to accommodate the use of plasmids with full-length HIV-1 genomes. HEK 293T cells were transfected as described above, and 16 h later the cells were seeded in the presence of a serial dilution of PIs. Virus supernatant was harvested 24 h later and used to infect fresh target HeLa-TZMbl cells by spinoculating for 2 h at 1200 ***g***. Replication was determined by measuring luciferase expression in infected target cells at 48 h post-infection using the SteadyGlo luciferase assay system (Promega) and expressed relative to that of no drug controls. Fifty per cent inhibitory concentrations (IC_50_) were determined using Prism GraphPad Software. The IC_50_ values calculated are the mean of at least two independent experiments.

### Flow cytometry

HIV-1-infected Jurkat cells were washed and fixed with 3 % paraformaldehyde (PFA), permeabilized in BD Perm Buffer (BD Biosciences) and stained with anti-HIV p24 monoclonal antibody conjugated to fluorescein isothiocyanate [HIV-1 p24 (24–4) FITC; Santa Cruz Biotechnology] to detect intracellular Gag. Acquisition was performed using a Becton Dickinson FACS Calibur and data were analysed using FlowJo software. Cells were used when >90 % were Gag positive.

### Quantitative real-time PCR assay to measure cell–free and cell–cell spread

To measure cell–cell transfer from an infected donor cell to an uninfected target cell, real-time PCR was used to detect *de novo* *pol*-transcripts as described in previous studies using this assay [10, 19, 37] with modifications to accommodate for the use of drug inhibitors (RTIs and PIs). Donor cells were infected at an m.o.i. of 0.3–0.5 with either HIV-1 NL4.3 (wild-type) or drug-resistant mutant virus by spinoculating at 2000 ***g*** for 2 h. Three days after infection, the donor cells were stained for Gag and analyzed by flow cytometry. Only donor cell cultures that were >90 % infected were used for experiments. This minimizes the background from spreading infection between donor cells after target cells are added to the culture. In total, 2×10^5^ pre-washed infected Jurkat cells (donors) per well on a 96-well plate were mixed with 8×10^5^ uninfected Jurkat cells (targets) per well in the presence of a serial dilution of the single inhibitor or combination of inhibitors being tested. The co-culture was incubated for 24 h at 37 °C after which the cells were pelleted, stored at −80 °C and genomic DNA was extracted (Qiagen). Quantitative real-time PCR was performed to measure cell–cell spread as described previously using primers and probes specific for HIV-1 *pol* DNA and the housekeeping gene albumin [10, 19, 37] (HIV-1 *pol* primers and probe: forward 5′-GTGCTGGAATCAGGAAAGTACTA-3′, reverse 5′-ATCACTAGCCATTGCTCTCCAATT-3′, probe 5′-TGTGATATTTCTCATGTTCATCTTGGGCCTTATCT-3′, albumin primers and probe: forward 5′-GCTGTCATCTCTTGTGGGCTGT-3′, reverse 5′-AAACTCATGGGAGCTGCTGGTT-3′, probe 5′-CCTGTCATGCCCACACAAATCTCTCC-3′).

Cell-free experiments were performed as previously described [19]. Briefly, pre-washed 2×10^5^ donor cells per well were allowed to produce virus over 24 h. Then, 100 µl of the virus supernatant was used to infect 1×10^6^ target cells per well by spinoculation at 2000 ***g*** for 2 h, in the presence of a serial dilution of the drug combination under investigation. Following infection by spinoculation, the target cells were incubated for 24 h, after which they were pelleted for total DNA extraction and subsequent real-time PCR quantification of infection as described previously.

### Drug combination studies

The real-time PCR-based infection assays described above were used for the drug combination studies. Antiretroviral agents from the RTI and PI classes were tested in clinically relevant combinations. The drugs were combined in a ratio based on the IC_50_ values of the individual drugs for cell-free infection. For example, if the IC_50_ of drug A=50 nM and the IC_50_ of drug B=100 nM, to test these drugs in combination, A+B were combined in a ratio of 1 : 2 [38]. Cell–cell and cell-free infection was assessed in the presence of a serial dilution of the combination and infection was determined by qPCR as described above. The percentage inhibition at each concentration was determined and expressed as a fraction of the ‘no-drug’ positive control. These values were used to determine the CI values for the drug combination using the drug synergy analysis software CompuSyn (Paramus). The CI obtained can then be interpreted to determine whether the interaction between the drugs under study is synergistic, additive or antagonistic. For the presentation of our data the following cut-offs for CI values according to Chou and Talalay are applied: CI <0.9 = synergy, CI 0.9–1.2 = addition, CI >1.2 = antagonism.
